# Assessment of Hygienic Practices in Beef Cattle Slaughterhouses and Retail Shops in Bishoftu, Ethiopia: Implications for Public Health

**DOI:** 10.3390/ijerph18052729

**Published:** 2021-03-08

**Authors:** Fanta D. Gutema, Getahun E. Agga, Reta D. Abdi, Alemnesh Jufare, Luc Duchateau, Lieven De Zutter, Sarah Gabriël

**Affiliations:** 1 Department of Microbiology, Immunology and Veterinary Public Health, College of Veterinary Medicine and Agriculture, Addis Ababa University, Addis Ababa 1000, Ethiopia; fanta.desissa@aau.edu.et; 2 Department of Veterinary Public Health and Food Safety, Faculty of Veterinary Medicine, Ghent University, 9820 Merelbeke, Belgium; lieven.dezutter@ugent.be; 3 Food Animal Environmental Systems Research Unit, Agricultural Research Service, U. S. Department of Agriculture, Bowling Green, KY 42101, USA; getahun.agga@usda.gov; 4 Department of Veterinary Biomedical Sciences, College of Veterinary Medicine, Long Island University, Greenvale, NY 11548, USA; Reta.Abdi@liu.edu; 5 Department of Animal Health, Alage Agricultural Technical Vocational Educational Training College, Ziway 1000, Ethiopia; alemju21@yahoo.com; 6 Department of Nutrition, Genetics and Ethology, Faculty of Veterinary Medicine, Ghent University, 9820 Merelbeke, Belgium; luc.duchateau@ugent.be

**Keywords:** hygienic practices, slaughterhouses, beef, retail shops, public health, Bishoftu

## Abstract

Understanding the potential drivers of microbial meat contamination along the entire meat supply chain is needed to identify targets for interventions to reduce the number of meatborne bacterial outbreaks. We assessed the hygienic practices in cattle slaughterhouses (28 employees) and retail shops (127 employees) through face-to-face interviews and direct personal observations. At the slaughterhouses, stunning, de-hiding and evisceration in vertical position, carcass washing and separate storage of offal were the identified good practices. Lack of hot water baths, absence of a chilling room, infrequent hand washing, insufficiently trained staff and irregular medical check-up were practices that lead to unhygienic handling of carcasses. At the retail shops, cleaning equipment using soap and hot water (81%), storing unsold meat in refrigerators (92%), concrete floors and white painted walls and ceilings were good practices. Adjacently displaying offal and meat (39%), lack of a cold chain, wrapping meat with plastic bags and newspapers, using a plastic or wooden cutting board (57%), infrequent washing of equipment and floors, and inadequately trained employees were practices that could result in unhygienic handling of beef. Our study identified unhygienic practices both at the slaughterhouses and retail shops that can predispose the public to meatborne infections, which could be improved through training and implementation of quality control systems.

## 1. Introduction

The global increase in human population is associated with an increased demand for foods of animal origin [1]. Consequently, ensuring the security, quality, and safety of food is a worldwide concern [2]. It is a particularly significant problem in developing countries as animals and products there are often produced under sub-optimal hygienic conditions [3,4].

Most of the meatborne bacterial outbreaks are usually attributed to contamination along the supply chain due to poor handling practices [5]. Food-producing animals are the major sources of many foodborne pathogens and can lead to meat contamination, which may result in a widespread occurrence of foodborne diarrheal illnesses in humans [6,7]. Cattle slaughterhouses are one of the critical units in the supply chain from which foodborne pathogens can disseminate along the processing and distribution continuum including retail shops subsequently reaching the consumers. As a result, good hygienic practices at slaughterhouses and during distribution to and storage at retail shops and during sales are key points in ensuring the quality and safety of meat to safeguard public health [8,9]. Inadequate facilities and improper handling of the animals at the slaughterhouses further aggravate the microbial contamination of beef which can result in the transmission of foodborne pathogens to humans [10,11].

Meat hygiene and safety is usually less controlled in many developing countries where meat for human consumption is approved based on visual inspection, if at all, without routine microbiological testing [11]. Several studies investigated the occurrence of pathogens along the entire beef supply chain [12,13,14,15,16], while others identified contamination at specific levels such as at slaughterhouses [17,18,19,20] and in retail shops [19,21,22] in different countries including Ethiopia. Contamination and cross-contamination from raw meat is a major cause of foodborne diseases particularly in developing countries [23,24]. According to World Health Organization estimation, foodborne diseases resulted in 600 million cases and 420,000 deaths resulting in nearly 33 million disability-adjusted life years globally with the highest mortality burden in Africa in 2010 [25]. Foods of animal origin such as beef are major contributors to the burden. The global burden of foodborne diseases due to all animal source foods and beef was estimated at 168 and 10 disability-adjusted life years per 100,000 population, respectively [26]. However, information on the burden of foodborne diseases due to poor meat handling practices is limited. Improving hygienic handling practices by meat handlers during meat production, distribution, storage and sales at retail shops prevent or reduce microbial contamination [8]. It is very evident that food safety problems require intervention measures along the entire beef supply chain. To identify specific targets for intervention in specified settings, a clear understanding of local drivers for microbial meat contamination along the meat production, processing, and distribution chain is needed.

In Ethiopia, there are over 300 local slaughterhouses that supply meat for local consumption with different capacities and facilities, however all with low basic hygienic standards [27]. Although foodborne bacteria have been reported from cattle at slaughterhouses and beef in the retail shops as reviewed by Abayneh et al. [13], little information is available concerning beef hygienic handling practices along the beef production and distribution continuum in Ethiopia. Therefore, the objective of this study was to assess beef hygienic handling practices at cattle slaughterhouses and retail shops to contribute to the identification of intervention targets.

## 2. Materials and Methods

### 2.1. Study Settings

This study was conducted from June 2017 to May 2018 at the two local cattle slaughterhouses (one municipal and one privately-owned) found in Bishoftu, and all 127 retail shops selling beef in Bishoftu town. The town is located in East Shoa Zone of Oromia region, Ethiopia. According to the 2007 Ethiopian census report [28], the total human population of Bishoftu town was estimated at 100,114. The slaughterhouses slaughtered cattle brought directly from open markets by retail shop owners. Both slaughterhouses were small in capacity where the municipal slaughterhouse and the private slaughterhouse usually slaughtered 5–15 and 15–30 cattle per day, respectively.

### 2.2. Study Design and Data Collection

Data were collected through face-to-face interviews and direct personal observation using pre-tested semi-structured questionnaires and checklists to assess the beef hygienic handling practices at slaughterhouses and beef retail shops (Supplementary Material). The questionnaires and checklist were adapted from similar previous studies conducted in Ethiopia [29,30] and structured into (i) sociodemographic characteristics of the respondents, (ii) check list for direct observations and, (iii) questions for face-to-face interviews. The questionnaires were first prepared in English and then translated into Afaan Oromo and Amharic, the commonly spoken local languages in the study area. Data were collected by three trained data collectors. All employees in the two slaughterhouses (municipal = 16 and private = 12) and one employee from each of the retail shops (*n* = 127) engaged in beef handling activities were included in the survey. The purpose of the study was explained to the study participants and data were collected after obtaining full written consent from the participants. At the end of each interview, completeness and accuracy of the data were checked and ensured by the principal investigator. Ethical clearance was obtained from College of Veterinary Medicine and Agriculture of Addis Ababa University, VM/ERC/06/05/09/2017), Ministry of Science and Technology of Ethiopia (Ref no.3/10/006/2018) and the University Hospital Gent, Belgium (Ref. no. 2017/0612).

### 2.3. Data Management and Analysis

The collected data were entered to Microsoft Excel spread sheet (Microsoft Corp., Redmond, Washington, DC, USA) and analysed using STATA version 15.1 (STATA Corp. College Station, TX, USA). Descriptive statistics such as frequency and percentage are used to summarize the data. Fisher’s exact test was used to assess the difference in the sociodemographic characteristics and hygienic handling practices of the employees between the municipal and private slaughterhouses. A *p*-value of less than 0.05 was set as a significance level. The hygienic handling practices at the beef retail shops were described descriptively.

## 3. Results

### 3.1. Hygienic Practices at Cattle Slaughterhouses 

#### 3.1.1. Sociodemographic Characteristics

Table 1 summarizes the sociodemographic characteristics of the employees at the municipal (*n* = 16) and private (*n* = 12) slaughterhouses. The private and the municipal slaughterhouses did not significantly differ based on the sex, age, level of education and main duty of their employees (Fisher’s exact test *p* > 0.05). However, there was a significant difference between the slaughterhouses with respect to years of experience of the employees (Fisher’s exact test *p* = 0.000). Employees at the municipal slaughterhouse had more years of work experience (mean = 9.8 years, standard deviation [SD = 5.2]) than those working in the private one (mean = 2.4 years, (SD =1.4)). The combined mean age of the employees from the two slaughterhouses was 32.3 years (SD = 8.1) ranging from 19–50 years. 

**Figure 1:**
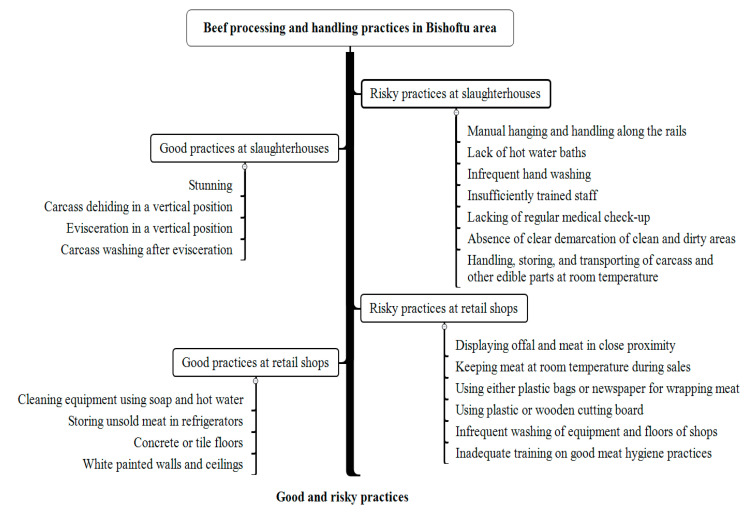
Beef processing and handling practices in the studied slaughterhouses and retail shops in Bishoftu town, Ethiopia.

Figure 1 summarizes the identified beef processing and handling practices in the two slaughterhouses and the beef retail shops evaluated in the study.

#### 3.1.2. Slaughter Process

Both slaughterhouses had their own veterinarian who was in charge of the supervision of slaughter process and meat inspection. Overall, the slaughter steps were similar at both slaughterhouses. The slaughtering started with the stunning of the animals by stabbing at the atlanto-occipital region using a sharp edge of knife, immediately followed by bleeding and removal of the head and the feet with the carcass in a horizontal position on the floor. The remaining slaughter steps (de-hiding, evisceration, post mortem inspection and carcass labeling) were performed in vertical position after manually hanging the carcass by hooks and sliding it over the rail system. Finally, the carcasses were stored and transported at room temperature.

#### 3.1.3. Beef Handling Practices

Both slaughterhouses reported the use of water from the municipal city supply. Hand washing was not a frequent practice during slaughter operations according to 53.6% of the respondents (Table 2, Figure 1). There was no significant difference between the municipal and private slaughterhouse based on hand washing practice, perceived sources of carcass contamination, training on meat hygiene and frequency of medical check-up of the employees (Fisher’s exact test *p* > 0.05). The use of aprons, white coats, boots and hair covering, as well as the presence of sinks for hand washing were good practices observed at both slaughterhouses. However, none of the employees wore hand gloves during operations. We also observed lack of hot water for hand washing and dipping of knives.

### 3.2. Beef Handling Practices at Retail Shops

#### 3.2.1. Sociodemographic Characteristics

The sociodemographic characteristics of the study participants from the retail shops are indicated in Table 3. All respondents (*n* = 127) were males with a mean age of 25.3 years (SD = 5.9) ranging from 18 to 56 years. Most (70.1%) respondents at retail shops attended only up to primary school and 85.8% of them did not receive training on the best practices of handling meat.

#### 3.2.2. Beef Handling Practices

According to the respondents, carcasses are transported from the slaughterhouses to the retail shops using closed vehicles without a cooling facility. The municipal water supply was the source of water for all retail shops. Of the retail shops, 39.4% displayed offal (heart, kidneys, liver, and stomach) and meat next to each other on the same display cabinet, 4.7% used the same knife for cutting offal and meat. Among the respondents, 85.0% of them used the same coat for the entire day; 9.0% did not wash hands before touching meat; 11.8% did not use soap for hand washing, and 2.4% collected money while handling meat. Ninety-two percent had a refrigerator for storage of leftover meat (Table 4). 

A variable frequency of washing equipment, display cabinet, and floor was reported. In most of the retail shops (>70%) equipment, floors and the display cabinet were cleaned once per day. The majority (81.1%) of retail shops reported cleaning their equipment with soap and hot water (Table 5). 

All respondents wore a white coat, but none of them put on gloves. In all retail shops, there were light bulbs, either concrete or tile floors and white painted walls and ceilings. However, in all shops meat was displayed at room temperature, with no covering, being exposed to dust particles and domestic flies. All shops used either plastic bags or newspapers for wrapping the meat (Figure 1). Among the retail shops, 85% had no hand wash sink at the display room. Standby hot water baths were not available for dipping knives. Unclean retail shop ceilings and white walls with observable dirty spots were noticed in about 79% of the shops. Table 6 summarizes the observational assessments on the hygienic status of the beef retail shops.

## 4. Discussion

Proper meat handling practices play a significant role in ensuring meat quality and safety [9]. Knowledge of meat hygienic handling practices during beef production, processing and distribution is essential to formulate preventive measures to mitigate the contribution of meat to foodborne diseases [31]. We investigated the status of beef hygienic handling practices in cattle slaughterhouses and retail shops in Bishoftu town, Ethiopia. Our study revealed both good and unhygienic handling practices at the slaughterhouses and retail shops. The discussion below focuses on the main meat handling practices identified with their potential implication for public health. Moreover, the practices are discussed in view of the requirements of the Ethiopian proclamations: Meat inspection proclamation (No. 274/1970) [32], Public health Proclamation (No. 200/2000) [33] and Food, Medicine and Health Care Administration and Control Proclamation (No. 661/2009) which enables controlling the safety and quality of food [34] and the Codex Alimentarius Commission (CAC) on general principles of food hygiene [35] and code of hygienic practice for meat [36] that have been formulated to ensure the production and marketing of sound, wholesome and quality meat and meat products for the consumer’s protection. Ethiopia is a member of the Codex Alimentarius Commission and the Codex standards are the basic reference materials for standard setting and serve as enforcing tools for food safety where there are no developed Ethiopian standards [37,38].

In the present study, lack of hot water baths for hand washing and dipping of knives, infrequent hand washing, insufficiently trained operational employees, lack of regular medical check-up and lack of cooling facilities were bad practices identified both at the slaughterhouses and retail shops. Hot water, which is essential for hand and knife washing to remove potential surface contaminants and to prevent further cross contamination of meat, was lacking at washing basins of both at slaughterhouses and retail shops [39]. Even though Ethiopia is a member of CAC, the present finding indicated lack of adherence to the requirements of CAC that demands the presence of an adequate and easily accessible supply of hot and cold potable water at all times during handling meat for effective sanitizing of equipment and hand washing [36].

According to 53.6% of the respondents at slaughterhouses hand washing was not a frequent practice during slaughter operations, and few (9.4%) employees at retail shops did not wash their hands before touching meat. This practice is not consistent with the requirements of the CAC which recommends that food handlers should wash their hands at every stage of food production to safeguard the consumer from foodborne diseases [35].

About 40% of slaughterhouses and 85.8% of retails shops employees did not receive training on hygienic handlings of meat. Previous studies also reported that a considerable proportion of meat processing employees [30,40,41] and meat retail shops employees [30,41] did not receive basic training on hygienic handling of meat. This is contrary to the basic requirements for personnel working in the food industry. Employees working in food establishments such as slaughterhouses and retail shops should be trained on food safety issues [42]. According to Food, Medicine and Health Care Administration and Control Proclamation (No. 661/2009) of Ethiopia, a certificate of competence from the appropriate organ is required for any person working in food catering [34]. The Food and Agriculture Organization (FAO) also recommends the provision of food safety training to food handlers as an important intervention to improve their knowledge and skills [43].

All employees at the slaughterhouses and 98% of the respondents at retail shops confirmed having had a medical check-up. However, when asked about the frequency of the check-up, answers were variable and not in line with the actual requirement by the Ethiopian regulatory body. Having a periodic medical check-up would partly limit the transmission of pathogens from sick or potentially carrier employees [44]. In addition, strict regulation in the uniformity of the frequency of the check-up as mentioned by the requirements of the Oromia Health Bureau—recommending the need for medical check-up of all employees in food establishments every three months—is essential.

Carcasses were stored at room temperature at the slaughterhouses and transported to beef retail shops using vehicles without cooling facilities. At all retail shops, meat was displayed openly with no cooling and no cover, being exposed to dust particles and domestic flies. The meat could remain as such for hours until sold. The mean annual temperature of the study area is estimated at 20.2 °C (range: 10.9–29.5 °C) [45], which is the ideal temperature suitable for the growth of a wide range of spoilage and pathogenic organisms to potentially unsafe levels. Cold chain management in meat storage and supply is an exceedingly important requirement to ensure the quality and safety of meat and meat products [46,47].

None of the employees in slaughterhouses and retail shops wore hand gloves during handling of meat. The use of gloves may protect the meat against contamination [48]. In countries where the frequent change of gloves is economically not feasible like in Ethiopia, frequent hand washing is an effective measure to prevent cross contamination of meat. 

At the slaughterhouses, the use of aprons, white coats, boots and hair covering, as well as the presence of sinks for hand washing were good practices observed at both slaughterhouses. These practices are important to protect both the personnel and the meat from exposure to pathogens [49]. 

Stunning of the animals, the hanging of carcasses over the rail system for dehiding and eviscerations, and carcass washing after eviscerations were good practices identified at the slaughterhouses. These practices are essential to ensure production of quality and safe meat and needs to be maintained at all times [32,33,34,35,36]. However, we observed that bleeding was carried out on the ground, and the hanging and de-hiding of the carcass were done manually. These operations can lead to carcass contamination from the ground, workers’ hands and cross contamination from carcass to carcass contact [43]. Automatic carcass hoisting, hide removal and sliding of carcasses reduces the risk of carcass contamination [20]. Establishing slaughterhouses equipped with the necessary facilities and basic infrastructures would improve the hygienic production in slaughterhouses particularly in government-based municipal slaughterhouses in Ethiopia.

According to the respondent’s perception, feces during evisceration, hides, handler’s hands and knifes were the potential sources of carcass contamination at the slaughterhouses whereby feces as well hides were identified as the major sources by 36% of them. This was consistent with previous reports [50,51]. Previous studies reported the occurrence of foodborne pathogens such as *E. coli* O157 and *Salmonella* in cattle feces and on hides and the possibility of their transfer to carcass during slaughter operations [52,53,54,55,56]. Further studies to identify all possible sources for carcass contamination and designing effective intervention measures are needed in these slaughterhouses. This would help to improve handling practices [57].

At retail shops, the use of soap and water for hand and equipment washing, storing leftover meat in refrigerators, concrete/tile made floors, and white painted walls and ceilings were the identified good practices. These were in line with the basic requirements of Ethiopian proclamations and can contribute to hygienic handling of meat [34,36]. However, displaying offal and meat in close proximity (39.4%), use of either plastic bags or newspapers for wrapping meat (53.5%), use of plastic or wooden cutting boards, use of one coat for the entire day (85%) and infrequent washing of equipment and floors were sub-standard practices that can lead to carcass contamination [34,36].

The use of plastic bags or newspapers were contrary to the requirements of the Ethiopian Food, Medicine and Healthcare Administration and Control Authority Proclamation (No. 661/2009) that require packaging material to be made out of substances, which are safe and suitable for their intended use, and the product to be packed in container which will safeguard its hygienic, safety, quality and food grade. Furthermore, the proclamation states that “no packaging material shall be put into use unless it complies with the international and national safety and quality standards”, which was lacking in the beef retails shops in Bishoftu town [34].

In most of the retail shops (>70%) equipment, floors and the display cabinet were cleaned once per day. Unclean retail shops ceilings and white walls with observable dirty spots were noticed in 79% of the shops. Frequent and scheduled cleaning of equipment and working environments at food establishments are the basic essential requirements to ensure the continuing effective control of food hazards likely to contaminate food [35].

In general, the observed unhygienic practices at the slaughterhouses and retail shops can be linked with lack or inadequate knowledge of basic hygienic practices [30,58,59,60], lack of infrastructure or facilities [61] and poor compliance to standards of good handling practices of food [59]. Moreover, the insufficient implementation of the government control systems and ensuing timely corrective actions by the food regulatory bodies, which is common in most developing countries including Ethiopia, might contribute to sustaining such unhygienic practices leading to a higher risk for human infection necessitating urgent interventions [4,56].

The study has some limitations. The study used questionnaires as a data collection tool, which relies totally on the answers of the respondents that might not necessarily correspond to the actual situation. For example, 91% of the employees at the retail shops and 46% of employees at slaughterhouses responded that they washed their hands before touching the meat and between activities during work, which was contrary to our observations. All the respondents confirmed having had a medical check-up. However, when asked about the frequency of the check-up, answers were variable and not in line with the actual requirement by the regulatory body. Combining questionnaires with personal observations reduced the study limitations in part, while of course, the presence of the study team might have induced practice changes.

## 5. Conclusions

The study showed a combination of good and unhygienic meat handling practices in slaughterhouses and retail shops. The unhygienic handling practices potentially lead to a higher possibility for contamination and cross-contamination of the meat and may have serious public health implications. The unhygienic handling practices coupled with consumption of raw or under cooked meat which is a common habit in Ethiopia [62,63] could serve as suitable pathways for meatborne pathogens to enter the food chain. Our findings suggest the need for interventions through provision of food safety training to improve hygienic meat handling practices along the beef supply chain. Improving the infrastructure of the slaughterhouses and retail shops and strengthening food quality control systems by the government regulatory authorities to verify the hygienic meat production and marketing at all stages needs more attention. Moreover, educational sessions such as information campaign to raise food handlers’ and consumers’ awareness of adequate cooking practices, kitchen hygiene, and personal hygiene are important intervention areas to ensure beef safety.

## Figures and Tables

**Table 1:** Sociodemographic characteristics of the slaughterhouses’ employees in Bishoftu town, Ethiopia.

Variables		Number (%) of Respondents (*n* = 28)
Sex	Male	25 (89.3)
	Female	3 (10.7)
Age	15–24	4 (14.3)
	25–54	24 (85.7)
Educational status	Informal	2 (7.1)
	Primary	12 (42.9)
	Secondary	10 (35.7)
	Higher education	4 (14.3)
Service duration in years	1–5	16 (57.1)
	>5	12 (42.9)
Main duty at the slaughterhouse	Stunning and bleeding	2 (7.0)
	De-hiding	18 (65.0)
	Evisceration	6 (21.0)
	Meat inspector	2 (7.0)

**Table 2:** Beef handling practices at slaughterhouses in Bishoftu town, Ethiopia.

Variables		Number (%) of Respondents (*n* = 28)
Hand washing between activities during work	Yes	13 (46.4)
	No	15 (53.6)
Perceived major source of carcass contamination	Feces during evisceration	10 (36.0)
	Hides	10 (36.0)
	Handler’s hand	2 (7.0)
	Knife	6 (21.0)
Received on the job training on meat hygiene practices	Yes	17 (60.7)
	No	11 (39.3)
Frequency of medical checkup	Every three months	14 (50.0)
	Every six months	14 (50.0)

**Table 3:** Sociodemographic characteristics of employees at retail shops in Bishoftu town, Ethiopia.

Variables		Number (%) of Respondents (*n* = 127)
Age	18–24	65 (51.2)
	25–56	62 (48.8)
Education level	Informal	9 (7.1)
	Primary	89 (70.1)
	Secondary	29 (22.8)
Ethnicity	Gurage	52 (40.9)
	Hadiya	28 (22.0)
	Oromo	21 (16.5)
	Amhara	18 (14.2)
	Tigire	8 (6.3)
Religion	Orthodox	82 (64.6)
	Protestant	45 (35.4)
Experiences in years	<5	88 (69.3)
	>5	39 (30.7)

**Table 4:** Respondent’s response on beef handling practices at retail shops in Bishoftu town, Ethiopia.

Variables		Number (%) of Respondents (*n* = 127)
Use of a clean white coat	Two per day	17 (13.4)
	One per day	108 (85.0)
	One every two days	2 (1.6)
Washing hands before touching meat	Yes	115 (90.6)
	No	12 (9.4)
Using of soap for hand washing	Yes	112 (88.2)
	No	15 (11.8)
Received training	Yes	18 (14.2)
	No	109 (85.8)
Medical checkup	Yes	125 (98.4)
	No	2 (1.6)
Frequency of medical checkups	Every three months	91 (71.6)
	Every six months	27 (21.3)
	Once per year	9 (7.1)
Fly control methods	Horsetail fly swatter	86 (68.0)
	Roach killer	4 (3.1)
	Fumigation	4 (3.1)
	Fumigation and roach killer	3 (2.4)
	Horsetail fly swatter and fumigation	3 (2.4)
	No control	27 (21.0)
Maximum duration of meat storage before sale	Two days	15 (11.8)
	One day	93 (73.2)
	12 h	19 (15.0)
Having refrigerator for storage	Yes	117 (92.1)
	No	10 (7.9)
Money collection from buyers by person handling the meat	Yes	3 (2.4)
	No	124 (97.6)
Storage of offal and meat on the same display cabinet	Yes	50 (39.4)
	No	77 (60.6)
Use of the same knife for offal and meat	Yes	6 (4.7)
	No	121 (95.3)
Is there a need for quality improvement?	Yes	3 (2.4)
	No	124 (97.6)
Complaint from consumers about the quality of meat	Yes	10 (7.9)
	No	117 (92.1)

**Table 5:** Equipment and floor washing practices at beef retail shops in Bishoftu town, Ethiopia.

Variables	Number (%) of Respondents (*n* = 127)
Frequency of washing equipment and floor	
Knife	
More than twice per day	9 (7.1)
Twice per day	9 (7.1)
Once per day	109 (85.8)
Cutting board	
More than twice per day	2 (1.6)
Twice per day	12 (9.4)
Once per day	113 (89.0)
Saw/axes	
Twice per day	4 (3.1)
Once per day	104 (81.9)
Once in every two days	19 (15.0)
Display cabinet	
Once per day	93 (73.2)
Every two days	34 (26.8)
Hooks	
Once per day	102 (80.3)
Every two days	25 (19.7)
Floor	
Once per day	89 (70.1)
Every Two days	38 (29.9)
Use of soap and hot water to clean equipment	
Yes	103 (81.1)
No	24 (18.9)

**Table 6:** Summary of the observational assessment of outcome of the hygienic status of the beef retail shops in Bishoftu town, Ethiopia.

Variables		Number (%) of Retail Shops (*n* = 127)
Floor-type	Tile	37 (29.1)
	Concrete	90 (70.9)
Clean wall and ceiling	Yes	27 (21.3)
	No	100 (78.7)
Presence of a sink for hand washing at the display	Yes	19 (15.0)
	No	108 (85.0)
Type of cutting board	Wood	33 (26.0)
	Marble	42 (33.1)
	Plastic	40 (31.5)
	Marble and plastic	8 (6.3)
	Marble and wood	4 (3.1)
Materials used for meat wrapping	Plastic bags	100 (79.0)
	News paper	7 (5.0)
	Plastic bags and news paper	20 (16.0)
Use of a head cover	Yes	51 (40.2)
	No	76 (59.8)

## Data Availability

Not applicable.

## Supplementary Materials

The following are available online at https://www.mdpi.com/1660-4601/18/5/2729/s1, Questions and check list to assess hygienic handling practices in slaughterhouses and in beef retailshops.
