# Supplementary material for: Assessment of Hygienic Practices in Beef Cattle Slaughterhouses and Retail Shops in Bishoftu, Ethiopia: Implications for Public Health
Source: Int J Environ Res Public Health. 2021 Mar 8;18(5):2729. doi: 10.3390/ijerph18052729 (PMC7967449; doi:10.3390/ijerph18052729)
Supplement: Supplementary file 1 [file ijerph-18-02729-s001.pdf]

I. Questions and check list to assess hygienic handling practices at slaughterhouses

A. Basic information

1. Date ___________
2. Code __________

B. Sociodemographic characteristics of the respondents

1. Sex: Male [ ] Female[ ]
2. Age: ________
3. Level of Education: Illiterate [ ] Informal Education [ ] Primary Education [ ] Secondary Education [ ]
   Other (Specify) ________________
4. Duration of working at the slaughter houses (in years)? ___________
5. Duty at the slaughter houses? Veterinarian/meat inspector [ ] Stunning [ ] De-hiding [ ]
   Other (specify) ________

C. Hygienic handling practices at slaughterhouses

C-1. Check list for observations

1. Stunning before slaughter: Yes[ ] No [ ]
2. Stunning method: _________________
3. Waiting time to start dehiding after stunning and bleeding: _______________
4. Method of carcass dressing: Vertical (hanging) Horizontal (on floor):
5. Presence of sink for washing hands in the slaughterhouse: Yes [ ] No [ ]
6. Carcass washing after evisceration: Yes [ ] No [ ]
7. Use of the following protective materials while working in the slaughterhouse:

Protective materials	Response	
	Yes	No
Apron		
white coat		
Head cover		
Gloves		
Boots		

C-2. Questions for face to face interviews

1. Do you wash your hands frequently during slaughter operation: Yes [ ] No [ ]
2. Do you wash your hands with soap: Yes [ ] No [ ]
3. Do you use the same knife for dehiding and evisceration: Yes [ ] No [ ]
4. Do you wash your hands in between activities: Yes [ ] No [ ]
5. Do you sink the knife in hot water after each activity: Yes [ ] No [ ]
6. What do you think is the possible sources for carcass contamination: [ ] Feces during evisceration [ ] hides during dehiding [ ] handlers hand [ ] knife [ ] floor [ ] hanging hook [ ] Others(specify)_________
7. What is the source of water used in the slaughterhouse: City/Municipal tap water [ ] borehole [ ] collected rain water [ ] River [ ] others (specify) _________________
8. Have you ever received any training on hygienic handling of meat: Yes [ ] No [ ]
9. Have you gone for medical checkups to work at the slaughterhouse: Yes [ ] No [ ]
10. If yes, how frequent you go for medical checkup: every three months [ ] every six months [ ] once per year [ ]
11. Do you think improvements are needed to avoid contamination of carcass at the slaughterhouse: Yes [ ] No [ ]
12. If yes, what kind of improvement: _______________________

II. Questions for face to face interview and check list for direct observation to assess hygienic handling practices at beef retailshops

A. Basic information

1. Date_______________
2. Code______________

B. Sociodemographic of characteristics

1. Sex: Male [ ] Female[ ]
2. Age____________
3. Level of Education: Illiterate [ ] Informal Education [ ] Primary Education [ ] Secondary Education [ ] Other (specify)____________
4. Duration of working at meat retail outlet (in years)? ______________
5. Religion______________
6. Ethnicity___________________

C. Hygienic handling practices at slaughterhouses

C-1. Check list for observations

1. Presence of any cover on meat display case: Yes [ ] No [ ]
2. Retail shop floor is made of : Concrete [ ] Tile [ ] Wood earthen material [ ] others(specify)_____
3. Wall and ceiling are clean or free of dust**:** Yes [ ] No [ ]
4. Wall painted with white color: Yes [ ] No [ ]
5. Ventilation status of display case and butchery: Good [ ] Fair [ ] Poor [ ]

   Good-ventilation allows air flow into the butchery but sieves off dust and other particles [ ]

   Fair-ventilation allows air flow but do not sieve dust or other particles or allows very little air flow [ ]

   Poor-ventilation does not allow air flow at all [ ]
6. Presence and use of bulbs at the display case: Yes [ ] No [ ]
7. Meat cooling facility (refrigerator) at the display cabinet: Yes [ ] No [ ]
8. Presence of sink for washing hands at beef sale point: Yes [ ] No [ ]
9. Type/kind of cutting board used: Wood [ ] plastic [ ] Metal [ ] concrete [ ] Marble [ ]
10. Presence of hot water baths for dipping knives: Yes [ ] No [ ]
11. Material used to pack or wrap meat for sale: Newspaper[ ] Plastic [ ] Used paper[ ]
12. Use the following protective materials while selling or handling meat:

Protective materials	Response	
	Yes	No
Apron/white coat		
Head cover		
Gloves		

C-2. Questions for face to face interviews

1. What is the means of transporting meat from slaughterhouse to the retail shop: Open vehicle [ ] Closed vehicle[ ] Animal transport (Cart horse) [ ]
2. How frequent do you use washed the protective coat (white coat and apron): Once per day in the evening [ ] Twice per day, morning and evening [ ] Once after every two days [ ] Once per week [ ] others [ ] ________
3. Do you have a refrigerator for storage of the meat that remains from daily sale: Yes [ ] No [ ]
4. Do you wash your hands before touching meat: Yes [ ] No [ ]
5. Do you wash your hands with soap: Yes [ ] No [ ]
6. What is the source of water for use in the butchery: City/Municipal tap water [ ] borehole [ ] rain collected water [ ] river [ ] others (specify) [ ]
7. How often do you wash the following butchery surfaces and equipment:

Frequency of washing	Equipments /surfaces					
	Knife	Cutting boards	Saw/Axes	Display cabinet	Hooks	Floors
Once per day in the morning						
Once per day in the evening						
Twice per day						
More than twice						
Once in every two days						
Others (specify)						

8. Do you use detergent/disinfectant for cleaning the butchery utensils: Yes [ ] No [ ]
9. If “Yes” what types of detergent/disinfectant:______________________
10. What is the way of cleaning butchery equipment: Using cold water only [ ], cold water with soap [ ] hot water only [ ] hot water with soap [ ] wiping with pieces of fabrics [ ] others (specify)_________
11. Is there routine control of flies at the retail shop: Yes [ ] No [ ]
12. If “Yes”, what is the method used to control flies: ______________
13. How long does the meat stay in your butchery before it is sold? Less than 12 hours [ ] One day [ ] Two days [ ]
14. Do you collect money while handling or selling meat: Yes [ ] No [ ]
15. Have you ever received any training on hygienic handling of meat: Yes [ ] No [ ]
16. Do you ever receive complaints from the consumers on the quality of the meat you sell: Yes [ ] No [ ]
17. If yes, what kind of complaint: Abdominal upsets [ ] Tough meat [ ] Dirty meat [ ] others [ ]________
18. Have you gone for medical checkups in the last 6 months: Yes [ ] No [ ]
19. How frequent you go for medical checkup: Every three months [ ] very six months [ ] Once per year [ ]
20. Do you have different storage and display cabinets for offal’s and meat: Yes [ ] No [ ]
21. Do you use the same equipment’s such as knife while handling meat and the offals: Yes [ ] No [ ]
22. Do you believe that the butchery where are you working requires some improvement for better handling of meat: Yes [ ] No [ ]
23. If yes, what kind of improvement:___________
